# Supplementary material for: Urbanisation generates multiple trait syndromes for terrestrial animal taxa worldwide
Source: Nat Commun. 2023 Aug 7;14:4751. doi: 10.1038/s41467-023-39746-1 (PMC10406945; doi:10.1038/s41467-023-39746-1)
Supplement: Supplementary file 1 — Supplementary Information [file 41467_2023_39746_MOESM1_ESM.pdf]

Supplementary Information for

Urbanisation generates multiple trait syndromes for terrestrial animal taxa worldwide

[†] These authors contributed equally, [*]Corresponding Authors: amy.hahs@unimelb.edu.au; bertrand.fournier@uni-potsdam.de; johan.kotze@helsinki.fi; marco.moretti@wsl.ch

Authors: Amy K. Hahs[†*1], Bertrand Fournier[†*2], Myla F. J. Aronson[3], Charles H. Nilon[4], Adriana Herrera-Montes[5], Allyson B. Salisbury[6], Caragh G. Threlfall[7,8], Christine C. Rega-Brodsky[9], Christopher A. Lepczyk[10], Frank A. La Sorte[11], Ian MacGregor-Fors[12], J. Scott MacIvor[13], Kirsten Jung[14], Max R. Piana[15], Nicholas S.G. Williams[1], Sonja Knapp[16,17,18], Alan Vergnes[19], Aldemar A. Acevedo[20], Alison M. Gainsbury[21], Ana Rainho[22], Andrew J. Hamer[23], Assaf Shwartz[24], Christian C. Voigt[25], Daniel Lewanzik[25], David M. Lowenstein[26], David O'Brien[27], Desiree Tommasi[28], Eduardo Pineda[29], Ela Sita Carpenter[30], Elena Belskaya[31], Gábor L. Lövei[32,33], James C. Makinson[34], Joanna L. Coleman[35], Jon P. Sadler[36], Jordan Shroyer[4], Julie Teresa Shapiro[37], Katherine C. R. Baldock[38,39,40], Kelly Ksiazek-Mikenas[41], Kevin C. Matteson[42], Kyle Barrett[43], Lizette Siles[44], Luis F. Aguirre[45], Luis Orlando Armesto[46], Marcin Zalewski[47], Maria Isabel Herrera-Montes[48], Martin K. Obrist[49], Rebecca K. Tonietto[50], Sara A. Gagné[51], Sarah J. Hinners[52], Tanya Latty[53], Thilina D. Surasinghe[54], Thomas Sattler[55], Tibor Magura[56,33], Werner Ulrich[57], Zoltan Elek[58], Jennifer Castañeda-Oviedo[59], Ricardo Torrado[60], D. Johan Kotze[†*,12], Marco Moretti[†*,61]

Institutes: [1]School of Agriculture, Food and Ecosystem Sciences, The University of Melbourne, Burnley Campus 500 Yarra Blvd, Richmond 3121 VIC Australia; [2]Institute of Environmental Science and Geography, University of Potsdam, Karl-Liebknecht-Str. 24-25, 14476 Potsdam, Germany; [3]Department of Ecology, Evolution and Natural Resources, Rutgers, The State University of New Jersey, New Brunswick, NJ 08816 USA; [4]School of Natural Resources, University of Missouri, Columbia, MO 65211 USA; [5]Department of Environmental Science, College of Natural Sciences, University of Puerto Rico, San Juan, Puerto Rico; [6]The Morton Arboretum, 4100 Illinois Route 53, Lisle, IL 60532, USA; [7]School of Life and Environmental Sciences, The University of Sydney, Sydney, NSW 2006, Australia; [8]School of Natural Sciences, Macquarie University, Sydney, NSW 2109, Australia; [9]School of Science and Mathematics, Pittsburg State University, Pittsburg, KS 66762 USA; [10]School of Forestry, Wildlife and Environment, Auburn University, Auburn, AL 36849, USA; [11]Cornell Lab of Ornithology, Cornell University, Ithaca, NY, 14850 USA; [12]Faculty of Biological and Environmental Sciences, Ecosystems and Environment Research Programme, University of Helsinki, Niemenkatu 73, FI-15140, Lahti, Finland; [13]Department of Biological Sciences, University of Toronto Scarborough, 1265 Military Trail, Toronto, M1C 1A4, Canada; [14]Institute of Evolutionary Ecology and Conservation Genomics, Ulm University, Albert-Einstein-Allee 11, 89069 Ulm, Germany; [15]USDA Forest Service, Northern Research Station, Amherst, MA 01002 USA; [16]Helmholtz Centre for Environmental Research – UFZ, Department of Community Ecology, Theodor-Lieser-Str. 4, 06120 Halle (Saale), Germany; [17]German Centre for Integrative Biodiversity Research (iDiv) Halle-Jena-Leipzig, Puschstraße 4, 04103 Leipzig, Germany; [18]Technische Universität Berlin, Department of Plant Ecology, Rothenburgstraße 12, 12165 Berlin, Germany; [19]CEFE, Univ Montpellier, CNRS, EPHE, IRD, Univ Paul Valéry Montpellier 3, Montpellier, France; [20]Departamento de Ciencias Ecológicas, Facultad de Ciencias, Laboratorio de Genética y Evolución, Universidad de Chile,

Las Palmeras 3425, Ñuñoa, Santiago, Chile; [21]University of South Florida, St. Petersburg Campus, Department of Integrative Biology, St. Petersburg, FL, 33701, USA; [22]cE3c – Centre for Ecology, Evolution and Environmental Changes at the Dept. of Animal Biology, Faculty of Sciences, Univ. of Lisbon, Lisboa, Portugal; [23]Institute of Aquatic Ecology, Centre for Ecological Research, Karolina u. 29, 1113 Budapest, Hungary; [24]Faculty of Architecture and Town Planning, Technion – Israel Institute of Technology, Haifa, 32000, Israel; [25]Dept. of Evolutionary Ecology, Leibniz Institute for Zoo and Wildlife Research, Alfred-Kowalke-Str. 17, 10315 Berlin, Germany; [26]Michigan State University Extension, Macomb County, 21885 Dunham Rd - Suite 12, Clinton Twp, MI 48036, USA; [27]Scottish Natural Heritage (NatureScot), Great Glen House, Inverness, IV3 8NW, UK; [28]Institute of Marine Sciences, University of California Santa Cruz, Santa Cruz, CA 95064, USA; [29]Red de Biología y Conservación de Vertebrados. Instituto de Ecología, A.C. Carretera Antigua a Coatepec 351, Xalapa, 91073, Mexico; [30]U.S. Fish and Wildlife Service, Chesapeake Bay Field Office, 177 Admiral Cochrane Dr. Annapolis, MD 21401, USA; [31]Institute of Plant and Animal Ecology, Ural Branch, Russian Academy of Sciences, Eighth March Street 202, Yekaterinburg 620144, Russia; [32]Department of Agroecology, Aarhus University, Flakkebjerg Research Centre, DK-4200 Slagelse, Denmark; [33]ELKH-DE Anthropocene Ecology Research Group, University of Debrecen, H-4032 Debrecen, Egyetem square 1, Hungary; [34]Hawkesbury Institute for the Environment, Western Sydney University, Locked Bag 1797, Penrith, NSW, 2751, Australia; [35]Queens College at the City University of New York, Flushing NY USA; [36]School of Geography, Earth and Environmental Sciences, University of Birmingham, Edgbaston, Birmingham B15 2TT, UK; [37]University of Lyon, French Agency for Food, Environmental and Occupational Health & Safety (ANSES), Laboratory of Lyon, 31 Avenue Tony Garnier, 69364, Lyon Cedex 07, France; [38]Department of Geography and Environmental Sciences, Northumbria University, Newcastle upon Tyne, UK; [39]School of Biological Sciences,

University of Bristol, Bristol, UK; [40]Cabot Institute, University of Bristol, Bristol, UK; [41]Department of Biology, Elmhurst University, Elmhurst, IL 60126 USA; [42]Department of Biology/Project Dragonfly, Miami University, Oxford, OH, USA; [43]Department of Forestry and Environmental Conservation, Clemson University, 261 Lehotsky Hall, Clemson, SC 29631, USA; [44]Área de Mastozoología, Museo de Historia Natural Alcide d'Orbigny. Avenida Potosí 1458, Cochabamba. Cochabamba, Bolivia; [45]Centro de Biodiversidad y Genética, Universidad Mayor de San Simón, c Sucre, frente Parque La Torre s/n, Cochabamba, Bolivia; [46]Tecnoacademia, CEDRUM, Servicio Nacional de Aprendizaje (SENA), Cúcuta, Colombia; [47]Museum and Institute of Zoology of the Polish Academy of Sciences, Wilcza 64, Warsaw 00-679, Poland; [48]Grupo de Ecologia Animal, Universidad del Valle, Cali, Colombia; [49]Swiss Federal Institute for Forest, Snow and Landscape Research WSL, Biodiversity and Conservation Biology, CH-8903 Birmensdorf, Switzerland; [50]Department of Natural Sciences, University of Michigan-Flint, 303 E Kearsley St., Flint, Michigan, 48502, USA; [51]University of North Carolina at Charlotte, 9201 University City Blvd., Charlotte, North Carolina, USA, 28223; [52]Department of City and Metropolitan Planning, University of Utah, Salt Lake City, Utah, USA; [53]Sydney Institute of Agriculture, School of Life and Environmental Sciences; University of Sydney, Sydney, Australia; [54]Department of Biological Sciences, Bridgewater State University, Bridgewater, MA 02325, USA; [55]Swiss Ornithological Institute, Seerose 1, CH-6204 Sempach, Switzerland; [56]Department of Ecology, Faculty of Science and Technology, University of Debrecen, H-4032 Debrecen, Egyetem square 1., Hungary; [57]Department of Ecology and Biogeography, Nicolaus Copernicus University, Lwowska 1, 87-100 Torun, Poland; [58]Centre for Agricultural Research, Plant Protection Institute, Eötvös Loránd Research Network, Herman Ottó út 15, Budapest 1022, Hungary; [59]Grupo de Investigación en Ecología y Biogeografía, Universidad de Pamplona, Pamplona, Colombia; [60]Secretaría de Educación del Municipio de

Cúcuta, Cúcuta, Colombia; [61]Swiss Federal Research Institute WSL, Biodiversity and Conservation Biology, Zürcherstrasse 111, 8903 Birmensdorf, Switzerland

Table of Contents

Supplementary Fig. 1: Global distribution of data included in this study

Supplementary Fig. 2: Additional information about functional diversity indices

Supplementary Fig. 3: Species accumulation curves for each taxonomic group

Supplementary Fig. 4: Correlations of diversity metrics across taxonomic groups

Supplementary Fig. 5: Correlations among environmental variables

Supplementary Table 1: Summary of the dataset used in the analyses

Supplementary Table 2: Summary of number of cities with different numbers of taxa sampled

Supplementary Table 3: Additional information about traits

Supplementary Table 4: Detailed description of **Amphibian** traits.

Supplementary Table 5: Detailed description of **Bat** traits.

Supplementary Table 6: Detailed description of **Bee** traits.

Supplementary Table 7: Detailed description of **Bird** traits.

Supplementary Table 8: Detailed description of **Carabid beetle** traits.

Supplementary Table 9: Detailed description of **Reptile** traits.

Supplementary Table 10: Factor loadings on global climate PCA axes

Supplementary References

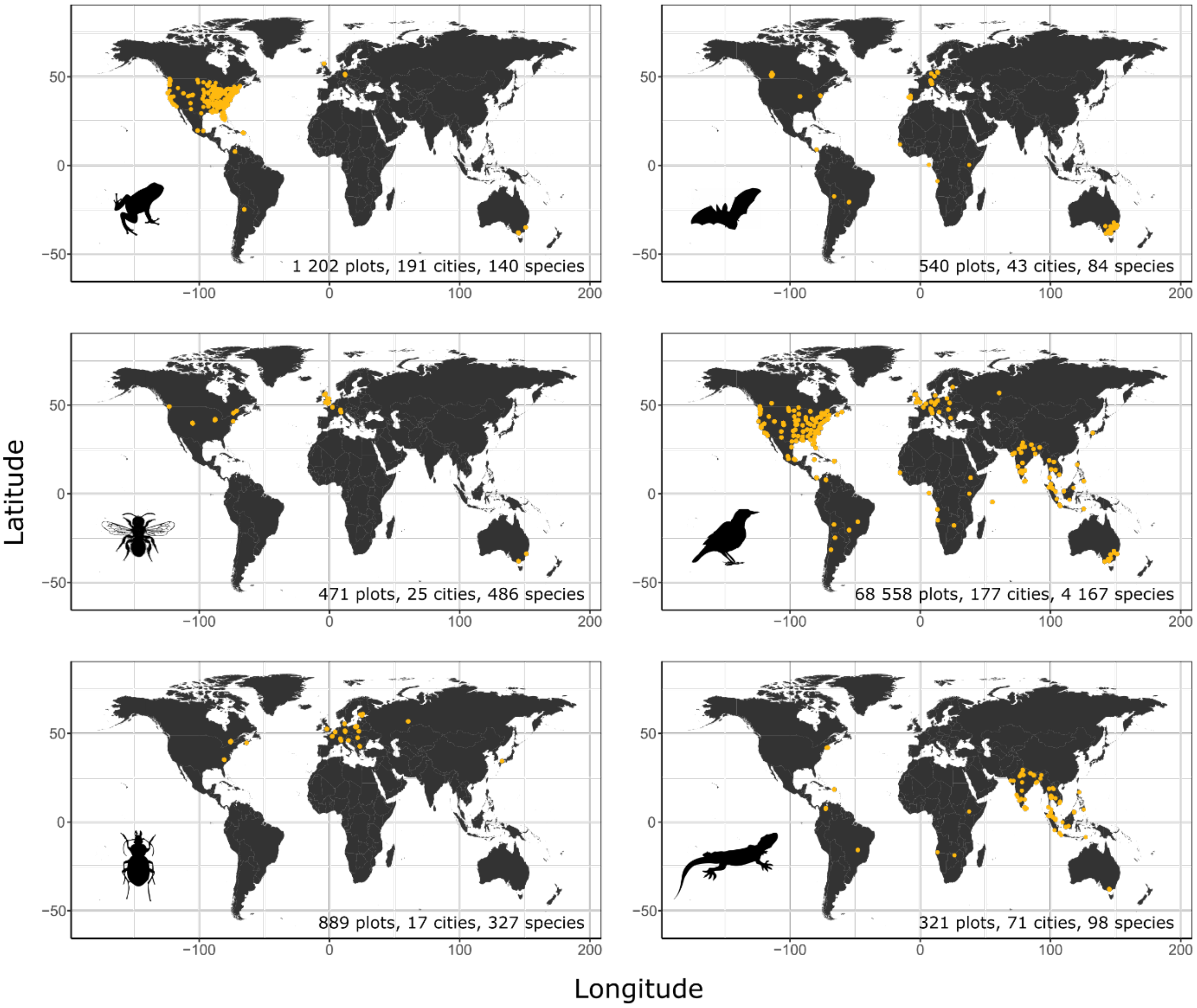

Supplementary Fig. 1: Global distribution of data included in this study. Locations of cities (orange dots) with sampling plots for each taxonomic group individually. All data come from the UrBioNet contributor network except for birds (eBird). Image credits: Ghedo and T. Michael Keesey (https://creativecommons.org/licenses/by-sa/3.0/) for the reptile. Michael Keesey (vectorization); Thorsten Assmann, Jörn Buse, Claudia Drees, Ariel-Leib-Leonid Friedman, Tal Levanony, Andrea Matern, Anika Timm, and David W. Wrase (photography) (https://creativecommons.org/licenses/by/3.0) for the carabid beetle. All other silhouette images come from www.phylopic.org and are public domain images.

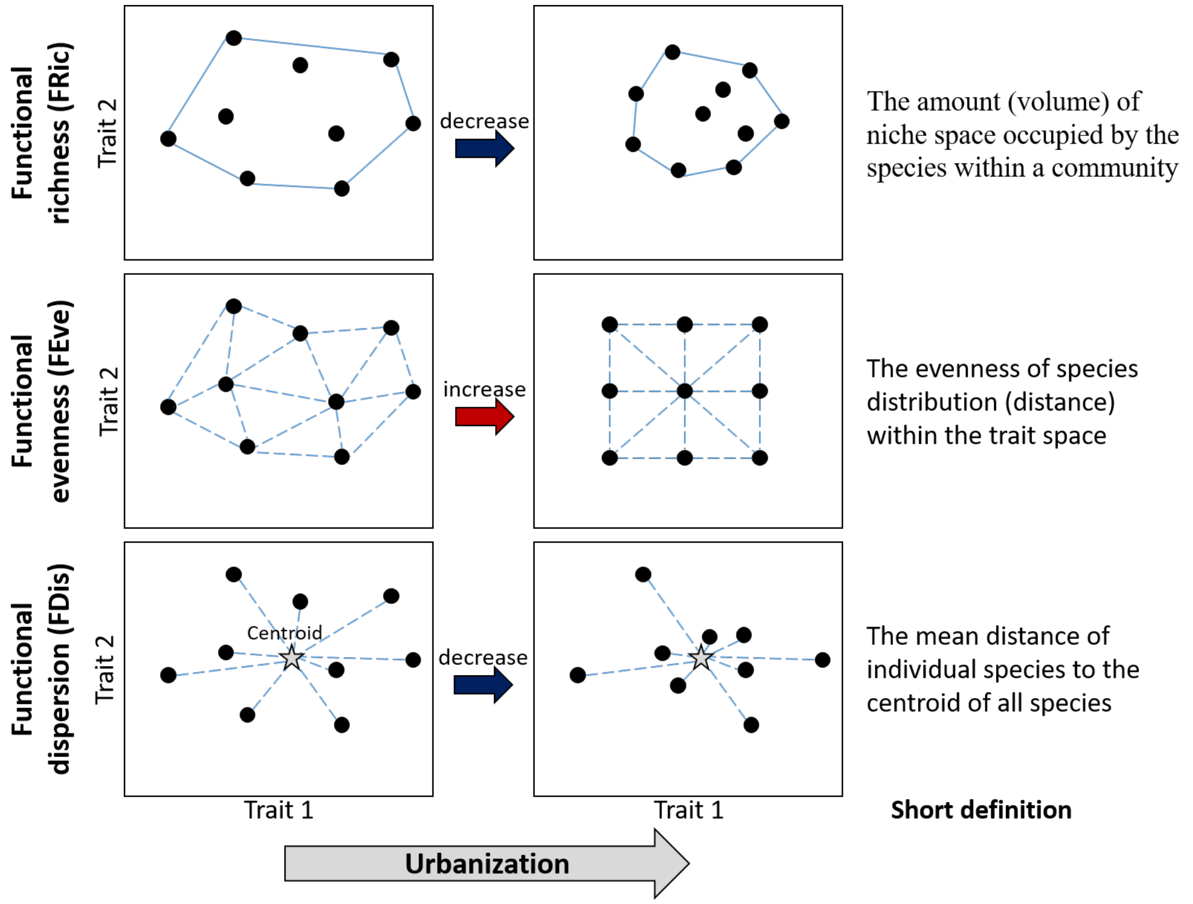

Supplementary Fig. 2. Additional information about functional diversity indices. Expected responses of functional richness (FRic), functional evenness (FEve) and functional dispersion (FDis) to increased urbanisation. Functional richness is expected to decrease as a result of the loss of some functional groups (environmental filtering). Functional evenness is expected to increase as a result of increased competition for more scarce resources (competitive exclusion of functionally similar species). Functional dispersion is expected to decrease because increased urbanisation is expected to select for generalist species with broad environmental tolerances (species close to the centroid). The right column provides a short definition of each index.

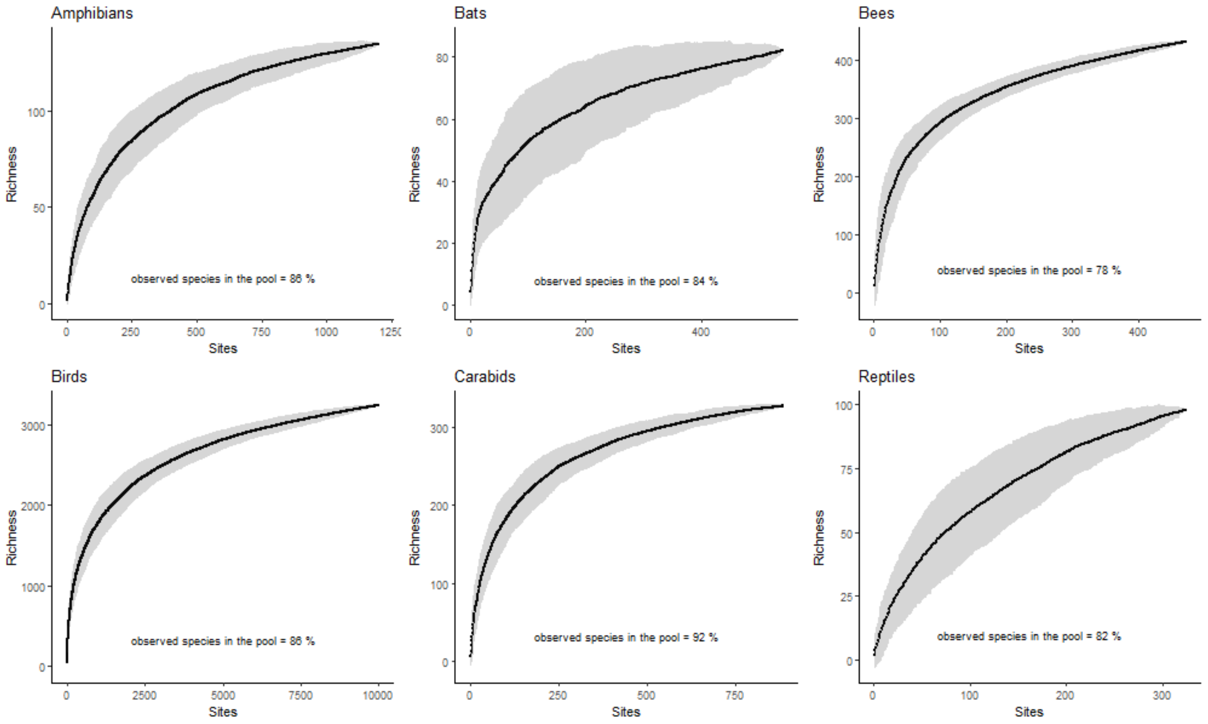

Supplementary Fig. 3. Species accumulation curves for each taxonomic group. These curves were used to estimate the total number of species present in the global species pool (extrapolated species richness in the species pool based on bootstrap resampling). Grey areas represent the variability in species richness estimates (± 2 standard deviation).

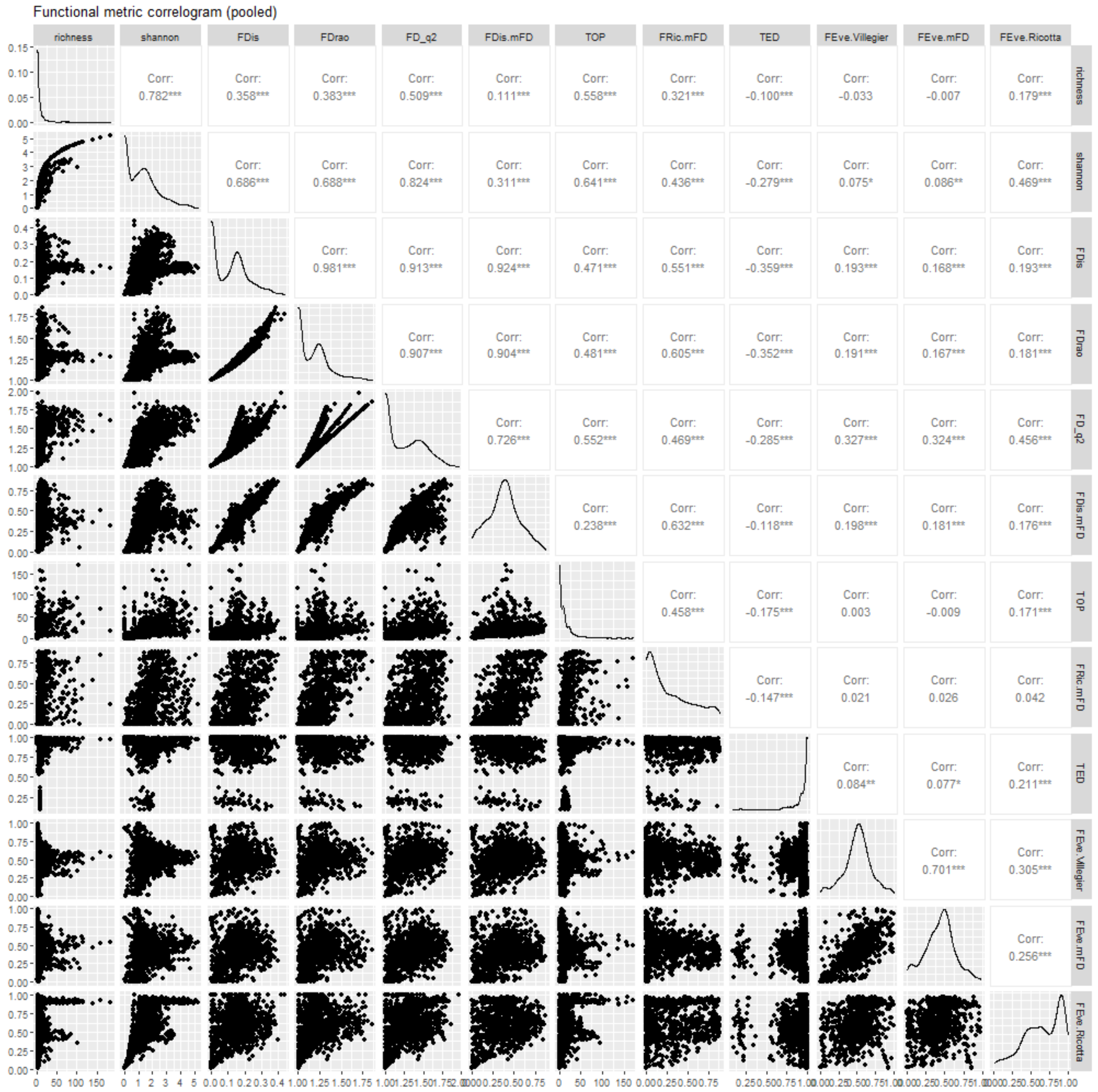

Supplementary Fig. 4. Correlations of diversity metrics across taxonomic groups. For each taxonomic group, 300 sites were randomly chosen and pooled to ensure that each group contributes equally to this analysis. For each functional diversity facet of interest, we selected the metric showing the lowest correlations to species richness (functional dispersion = FDis_mFD, richness = FRic_mFD, evenness = FEve_mFD). Stars indicate significant correlations (* $p < 0.05$; ** $p < 0.01$; *** $p < 0.001$).

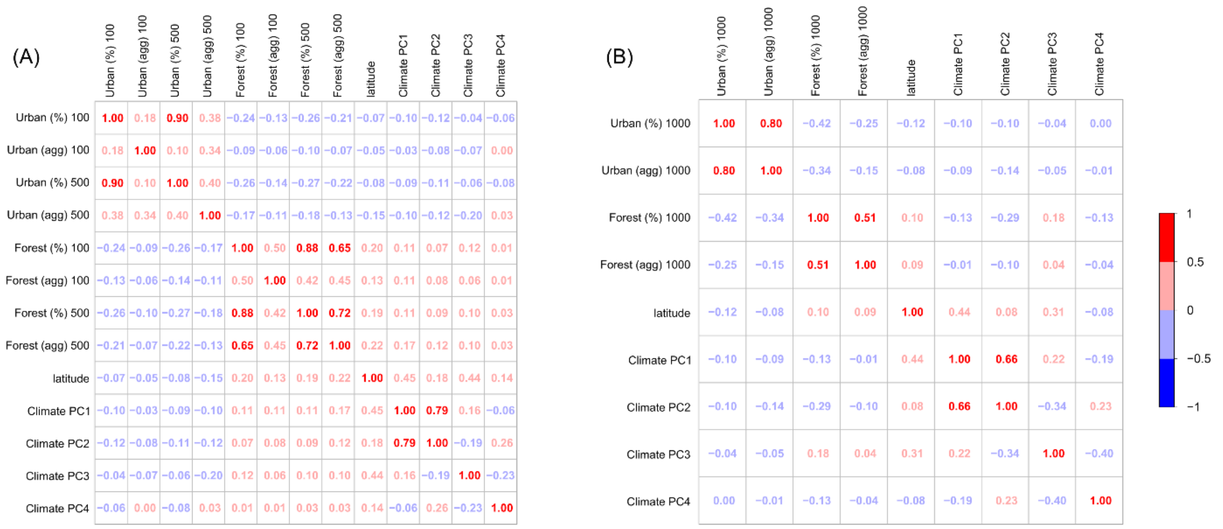

Supplementary Fig. 5: Correlations among environmental variables. A = all taxa except birds; B = birds. Correlations between predictors are relatively low between urban land cover, forest land cover, latitude, and climate while being relatively high between percent cover and aggregation, as well as among different scales. Blue = positive correlations; Red = negative correlations. Bolded values indicate significant correlations ($p < 0.05$).

Supplementary Table 1: Summary of the dataset used in the analyses and whether the data were compiled from directly contributed datasets, or e-Bird. The geographical distribution of the sampling plots for each taxonomic group is shown in Fig. 1 and Supplementary Fig. 1.

Taxa	***N. Plots***	***N. Cities***	***N. Species***	***Source***
Amphibians	1 202	191	140	UrBioNet contributor network
Bats	540	43	84	UrBioNet contributor network
Bees	471	25	486	UrBioNet contributor network
Birds	68 558	177	4 167	e-Bird
Carabids	882	17	327	UrBioNet contributor network
Reptiles	324	71	98	UrBioNet contributor network

Supplementary Table 2: Summary of number of cities with different numbers of taxa sampled. For example, only one city has been sampled for 5 taxa (Melbourne, Australia), and 3 cities have been sampled for 4 taxa (Lugano, Luzern and Zürich, Switzerland). The geographical distribution of the sampling plots for each taxonomic group is shown in Fig. 1 and Supplementary Fig. 1.

Number Taxa Sampled in the City	Number of Cities
1	254
2	109
3	12
4	3
5	1

Supplementary Table 3: Information about the evaluated traits presented in Fig. 2 of the manuscript. Specific traits are presented here but have not been shown in Fig. 2 of the manuscript. Further information around these traits and the data sources used for each individual taxonomic group can be found in Supplementary Tables 4-9.

Taxonomic group	***Body size***	***Feeding***	***Mobility***	***Reproductive Strategy***	***Specific traits***
Amphibians	**Body length** [cm]	**Diet breadth** (specialist=0; generalist=1)	**Movement Distances** (reduced=0; moderate=1; high=2)	**Clutch size** (0=small; 1 = intermediate; 2=large)	Aquatic habitat affinity index (0=low;1=medium; 2=high)
Bats	**Forearm length** [mm]	**Hunting strategy** (gleaning=1; others=0)	**Aspect ratio**	**Roosting requirements** (specialist=0; generalist=1)	Wing loading (nb) / Echolocation (kHz) / Dispersal strategy (mobility in open habitats=1; others=0)
Bees	**Inter-tegula distance** [mm]	**Tongue length** (short tongue=1; long tongue=0)	**Inter-tegula distance** [mm]	**Sociality** (Solitary =1; other=0)	Nesting strategy (Below ground (Below ground =1; others=0) / Above ground (Above ground =1; others=0) / Parasite (Parasite=1; others=0))

Birds	**Body mass** [g]	Trophic niche (**omnivorous**=1; others=0 / Fruit-nectar=1; others=0 / Invertebrate=1; others=0 / Plant-seed=1; others=0 / Vertebrates-scavenger =1; others=0)	**Hand-wing index**	**Clutch size** (number of eggs)	Foraging strata index (Habitat [0=aquatic; 1=terrestrial; 2=aerial]; Aquatic [0-2]; Terrestrial [0-4], Aerial [0-1])
Carabids	**Body length** [cm]	Trophic guild (Herbivore=1; others=0, Carnivore= 1; others=0, Omnivore=1; others=0)	**Wing morphology** (0=brachypterous; 1=dimorphic; 2=macropterous)	**Overwintering strategy** (imago hibernator=1; others=0)	Abiotic tolerance (0=hygro-; 1=meso-; 2=xerophilous)
Reptiles	**Body length** [cm]	**Diet breadth** (specialist=0, generalist=1)	**Movement distances** (reduced=0; moderate=1; high=2)	**Clutch size** (0=small; 1 = intermediate; 2=large)	Aquatic habitat affinity index (0=low;1=medium; 2=high)

Supplementary Table 4: Detailed description of **Amphibian** traits.

Trait	*Description and unit*	*Trait type*	*Sources*
Body size	**Mean body length** (in cm) from the tip of the snout to the most posterior opening of the cloacal slit, snout–vent length (SVL). In the case of salamanders, total length measurements include body and tail.	Continuous	Baker et al. 2011[1]<br>Beebee & Griffiths 2000[2]<br>Frost 2021[3]<br>Lips et al. 2003[4]<br>Stevens et al. 2014[5]<br>Trochet et al. 2014[6]<br>amphibiaweb.org<br>animaldiversity.org<br>iucnredlist.org<br>research.amnh.org
Mobility	**Mobility.** Three categories: reduced (≤ 100 m), moderate (101 – 1000 m), and high (> 1000 m) levels of mobility in relation to regional pools.	Semi-continuous (0, 1, 2)	
Reproductive strategy	**Clutch size.** Three categories: small clutches (≤ 20 eggs), medium (21 – 300 eggs), and large (> 300 eggs).	Semi-continuous (0, 1, 2)	
Feeding	**Diet.** Two categories: specialists (those who ingest 1-2 food types), and generalists (consuming 3 or more food types). When this information is not available, use mouth size as a proxy of feeding traits, with larger mouths representing generalist species and smaller mouths representing specialists.	Semi-continuous (0, 1)	
Taxon specific	**Aquatic index.** Three categories: exclusively terrestrial, occupying ponds or multiple habitats, or exclusively riparian.	Semi-continuous (0, 1, 2)	Expert knowledge for single species scarcely documented.

Supplementary Table 5: Detailed description of **Bat** traits.

Trait	***Description and unit***	***Trait type***	***Sources***
Body size	**Forearm length** (in mm)	Continuous	Denzinger & Schnitzler 2013[7]<br>Jung & Threlfall 2018[8]<br>Expert knowledge for single species scarcely documented.
Mobility	Aspect ratio: the **ratio of wing span to wing area**. Higher aspect ratio enables fast, but less manoeuvrable flight.	Continuous	
Reproductive strategy	Bats were grouped into species specialized on certain **roosting requirements** (e.g., caves, foliage) or those that are flexible in their choice of roosting sites.	Categorical	
Feeding	Species were classified as those catching aerial insects in flight (**aerial hunters**) **and others**, which include gleaning prey from surfaces or the vegetation (gleaning), or perch hunting (the latter two categories were not abundant enough to keep separate and hence were merged for analysis).	Categorical	
Taxon specific	**Wing loading**: wing area per body mass<br>**Echolocation** (kHz): frequency of maximum amplitude or characteristic frequency (in the case of zero-cross-based recordings, i.e. Anabat recording systems) of echolocation calls.<br>**Habitat preference** classified as **foraging** in open habitats, or edge or cluttered habitats. The latter two were grouped due to insufficient numbers of species. The two categories were: foraging in open space=1; and others=0 (clutter, edge space).	Continuous<br>Continuous<br>Categorical (0,1)	

Supplementary Table 6: Detailed description of **Bee** traits.

Trait	***Description and unit***	***Trait type***	***Sources***
Body size	Body size was given using the **inter-tegula distance**, ITD (in mm), given the two measures are highly correlated. ITD is the space between the two tegulae, which are the insertion points for each forewing. ITD measurements were obtained from the authors of each study, and are usually measured using an ocular micrometer or handheld calipers.	Continuous	Hinners et al. 2012[9]<br>Normandin et al. 2017[10]<br>Threlfall et al. 2015[11]<br>Cariveau et al. 2016[12]<br><br>Expert knowledge for single species scarcely documented.
Mobility	**Inter-tegula distance**, ITD (mm) as above.	Continuous	
Reproductive strategy	**Sociality** was used as a proxy for reproductive strategy since it integrates several reproduction features (e.g., number of brood cells, gender organisation etc.). We classified sociality as 'solitary' and 'other', where the latter included eusocial, primitively-social or semi-social.	Categorical	
Feeding	**Tongue length,** categorised as short or long mouthparts. If species data were missing, tongue length was estimated using bee family and inter-tegula distance as per Cariveau et al. (2016) [12], and subsequently assigned as short or long.	Categorical	
Taxon specific	Bees use a diversity of nesting locations or substrates, some of which can be heavily impacted upon by features of the urban environment. To simplify across the various **nesting strategies** that have been documented (Michener 2000[13]) we classified species to the following:<br><br>Below ground (Below ground=1; others=0) Above ground (Above ground=1; others=0) Parasite (Parasite=1; others=0)	Categorical	

Supplementary Table 7: Detailed description of **Bird** traits.

Trait	***Description and unit***	***Trait type***	***Sources***
Body size	Geometric mean of **body mass** average values for both sexes [in g].	Continuous	Jetz et al. 2008[14]<br>Sheard et al. 2020[15]<br>Wilman et al. 2014[16]
Mobility	**Hand-wing index**, ratio of the difference between wing length (from carpal joint to tip of longest primary feather) and secondary length (from carpal join to tip of 1st secondary feather) by wing length [(wl-sl)/wl].	Continuous	
Reproductive strategy	**Clutch size** [average number of laid eggs per nest].	Continuous	
Feeding	Categorical **diet** assigned based on the dominant among five diet categories, based in the summed scores of individual diets [fruit-nectar (e.g., fruits, drupes, nectar, pollen, plant exudates, gums), invertebrates (e.g., shrimp, krill, crustaceans, molluscs, cephalopods, gastropods, insects, worms, etc.), plant-seed (e.g., seeds, nuts, grains, and other plant materials not included in fruit-nectar), vertebrates-scavenger (e.g., vertebrates, carrion, garbage, etc.), omnivorous (score of ≤ 50 of all specific categories)].	Categorical	
Taxon specific	**Foraging strata index**. *Habitat* [0=aquatic; 1=terrestrial; 2=aerial] = (below surface + around surface) + 2*(ground + understory + mid high + canopy) + 3*(aerial);<br><br>*Aquatic*: [0 = does not forage in aquatic systems, 1 = forage on or just below water surface (<12.7cm), 2 = forage below water surfaces] = below surface + 2*around surface;<br><br>*Terrestrial* [0=does not feed in terrestrial systems, 1=feed on the ground, 2=feeds on the understory below 2 m, 3=feeds between 2 m and tree canopy, 4=feeds in the tree	Semi-continuous and categorical	

	canopy] = ground + 2* understory + 3 * mid high + 4 *canopy.<br><br>*Aerial* [0=does not feed well above vegetation or any structures, 1=feed well above vegetation or any structures].		

***Supplementary Table 8**:* Detailed description of **Carabid beetle** traits.

Trait	***Description and unit***	***Trait type***	***Sources***
Body size	Mean **body length** from the tip of the head to the tip of the abdomen (in mm)	continuous	Klaiber et al. 2017[17]<br><br>Lindroth 1985[18], 1986[19]<br><br>carabids.org
Feeding	Trophic guild. Three categories: *herbivore*, *carnivore,* ***omnivore***.	Categorical (0=no, 1=yes)	
Mobility	**Hind wing development**. Three categories: *brachypterous* (short-winged or wingless), *dimorphic* (short and long-winged individuals present in the same species), *macropterous* (long-winged).	Semi-continuous (0, 1, 2)	
Reproductive strategy	**Overwintering strategy**. Two categories: *spring breeder* (imago/adult hibernators, these species reproduce in the spring to early summer, their larvae develop in the summer and a new adult generation appears in the autumn, with these adults overwintering); *autumn breeder* (larval hibernators – these species reproduce in the summer or autumn and overwinter as larvae).	categorical (y/n)	
Taxon specific: Drought tolerance	**Tolerance to drought conditions**. Three categories: *hygrophilic* (wetness preference), *mesophlic* (intermediate preference) and *xerophilic* (drought preference).	Semi-continuous (0, 1, 2)	

Supplementary Table 9: Detailed description of **Reptile** traits.

Trait	***Description and unit***	***Trait type***	***Sources***
Body size	Total **body length** for lizards, snakes, and crocodiles. **Carapace length** for turtles (in cm).	continuous	Stevens et al. 2014[5]<br>reptile-database.org<br>animaldiversity.org<br>iucnredlist.org<br>research.amnh.org<br>Expert knowledge for single species scarcely documented.
Mobility	**Mobility.** Three categories: 0=reduced (≤ 100 m), 1=moderate (101 - 1000 m), and 2=high (> 1000 m) levels of mobility in relation to their year-round activities.	Semi-continuous (0, 1, 2)	
Reproductive strategy	**Clutch size.** Three categories: 0=small clutches (≤ 20 eggs), 1=medium (21 – 100 eggs), and 2=large (> 100 eggs).	Semi-continuous (0, 1, 2)	
Feeding	**Diet.** Two categories: specialists (those who ingest 1-2 food types), and generalists (consuming 3 or more food types). When this information is not available, use mouth size as a proxy of feeding traits, with larger mouths representing generalist species and smaller mouths representing specialists.	Semi-continuous (0, 1)	
Taxon specific:	**Aquatic index.** Three categories: 0=exclusively terrestrial, 1=occupying ponds or multiple habitats, or 3=exclusively riparian.	Semi-continuous (0, 1, 2)	

Supplementary Table 10: Factor loadings on global climate PCA axes. Only the first four axes that were retained for further analyses are shown. PC1 = cold-warm temperature; PC2 = broad (e.g. deserts) – narrow diurnal range (e.g. tropics); PC3 = high-low variability of temperatures; PC4 = high-low seasonality of precipitation.

	**PC1 (55%)**	**PC2 (19%)**	**PC3 (9%)**	**PC4 (6%)**
clim01: Annual Mean Temperature	**-0.284**	0.197	0.049	-0.063
clim02: Mean Diurnal Range	-0.137	**0.401**	0.084	0.009
clim03: Isothermality	-0.270	0.032	-0.264	-0.007
clim04: Temperature Seasonality	0.223	0.096	**0.479**	0.125
clim05: Max Temperature of Warmest Month	-0.253	0.267	0.185	-0.031
clim06: Min Temperature of Coldest Month	**-0.296**	0.121	-0.089	-0.094
clim07: Temperature Annual Range	0.174	0.216	**0.512**	0.141
clim08: Mean Temperature of Wettest Quarter	-0.248	0.223	0.235	0.055
clim09: Mean Temperature of Driest Quarter	-0.271	0.150	-0.140	-0.152
clim10: Mean Temperature of Warmest Quarter	-0.259	0.249	0.180	-0.037
clim11: Mean Temperature of Coldest Quarter	**-0.294**	0.141	-0.084	-0.082
clim12: Annual Precipitation	-0.249	-0.281	0.092	0.151
clim13: Precipitation of Wettest Month	-0.245	-0.193	0.014	**0.370**
clim14: Precipitation of Driest Month	-0.163	**-0.325**	0.269	-0.239
clim15: Precipitation Seasonality	0.030	0.150	-0.200	**0.677**
clim16: Precipitation of Wettest Quarter	-0.246	-0.198	0.016	**0.362**
clim17: Precipitation of Driest Quarter	-0.166	**-0.327**	0.265	-0.234
clim18: Precipitation of Warmest Quarter	-0.192	-0.205	**0.287**	0.237
clim19: Precipitation of Coldest Quarter	-0.185	-0.266	0.001	0.015

Supplementary References

1. Baker, J. *et al. Amphibian habitat management handbook*. Amphibian and Reptile Conservation, Bournemouth, vol. 39. (2011)
2. Beebee, T. J. C. & Griffiths, R. A. *Amphibians and reptiles: A Natural History of the British Herpetofauna*. The new naturalist, Collins, London, p. 45–56. (2000)
3. Frost, D. R. Amphibian species of the world: an online reference, version 5.4. American Museum of Natural History. http://research.amnh.org/vz/herpetology/amphibia. (2021)
4. Lips, K. R., Reeve, J. D., & Witters, L. R. Ecological traits predicting amphibian population declines in Central America. *Conserv. Biol.***17(4),** 1078-1088. (2003).
5. Stevens, V. M. *et al.* A comparative analysis of dispersal syndromes in terrestrial and semi-terrestrial animals. *Ecol. Lett.***17**, 1039–1052. (2014)
6. Trochet, A. *et al.* A database of life-history traits of European amphibians. *Biodiver. Data J.* **2**. (2014)
7. Denzinger, A. & Schnitzler, H. U. Bat guilds, a concept to classify the highly diverse foraging and echolocation behaviors of microchiropteran bats. *Front. Physiol.* **4**, 164. (2013)
8. Jung, K. & Threlfall, C. G. Trait-dependent tolerance of bats to urbanization: a global meta-analysis. *Proc Roy. Soc. B,* **285(1885),** p. 20181222. (2018)
9. Hinners, S. J., Kearns, C. A. & Wessman, C. A. Roles of scale, matrix, and native habitat in supporting a diverse suburban pollinator assemblage. *Ecolog. Applic.* **22**, 1923–1935. (2012)
10. Normandin, E. *et al.* Taxonomic and functional trait diversity of wild bees in different urban settings. *PeerJ* **5**, e3051. (2017)
11. Threlfall, C. G. *et al.* The conservation value of urban green space habitats for Australian native bee communities. *Biolog. Conserv.* **187**, 240–248. (2015)
12. Cariveau, D. P. *et al.* The allometry of bee proboscis length and its uses in ecology. *PloS one*, **11**, e0151482. (2016)
13. Michener, C. D. *The bees of the world*. Johns Hopkins University Press, Baltimore., MD. (2000)
14. Jetz, W., Sekercioglu, C.H.& Böhning-Gaese, K. The worldwide variation in avian clutch size across species and space. *PLoS biology*, **6**, e303. (2008)
15. Sheard, C. *et al.* Ecological drivers of global gradients in avian dispersal inferred from wing morphology. *Nat. Commun.***11**, 2463. (2020)
16. Wilman, H. *et al.* EltonTraits 1.0: Species-level foraging attributes of the world's birds and mammals: Ecological Archives E095-178. *Ecology*, **95**, 2027. (2014)
17. Klaiber, J. *et al. Fauna Indicativa*. WSL Berichte 54 : 198 S. (2017)

18. Lindroth, C. H. *The Carabidae (Coleoptera) of Fennoscandia and Denmark.* Fauna Entomologica Scandinavica 15, part 1. Scandinavian Science Press Ltd, Copenhagen, Denmark. Brill Archive (1). (1985)

19. Lindroth, C. H. *The Carabidae (Coleoptera) of Fennoscandia and Denmark*. Fauna Entomologica Scandinavica 15, part 2. Scandinavian Science Press Ltd, Copenhagen, Denmark. Brill Archive (1). (1986)
