# Supplementary material for: Urbanisation generates multiple trait syndromes for terrestrial animal taxa worldwide
Source: Nat Commun. 2023 Aug 7;14:4751. doi: 10.1038/s41467-023-39746-1 (PMC10406945; doi:10.1038/s41467-023-39746-1)
Supplement: Supplementary file 3 — Reporting Summary [file 41467_2023_39746_MOESM3_ESM.pdf]

nature portfolio

Corresponding author(s): Amy Hahs

Last updated by author(s): 2023-05-26

Reporting Summary

Nature Portfolio wishes to improve the reproducibility of the work that we publish. This form provides structure for consistency and transparency in reporting. For further information on Nature Portfolio policies, see our Editorial Policies and the Editorial Policy Checklist.

Please do not complete any field with "not applicable" or n/a. Refer to the help text for what text to use if an item is not relevant to your study.
For final submission: please carefully check your responses for accuracy; you will not be able to make changes later.

Statistics

For all statistical analyses, confirm that the following items are present in the figure legend, table legend, main text, or Methods section.

n/a	Confirmed	
☐	☑	The exact sample size (*n*) for each experimental group/condition, given as a discrete number and unit of measurement
☐	☑	A statement on whether measurements were taken from distinct samples or whether the same sample was measured repeatedly
X	☐	The statistical test(s) used AND whether they are one- or two-sided<br>*Only common tests should be described solely by name; describe more complex techniques in the Methods section.*
☐	☑	A description of all covariates tested
☐	☑	A description of any assumptions or corrections, such as tests of normality and adjustment for multiple comparisons
☐	☑	A full description of the statistical parameters including central tendency (e.g. means) or other basic estimates (e.g. regression coefficient) AND variation (e.g. standard deviation) or associated estimates of uncertainty (e.g. confidence intervals)
X	☐	For null hypothesis testing, the test statistic (e.g. *F*, *t*, *r*) with confidence intervals, effect sizes, degrees of freedom and *P* value noted<br>*Give P values as exact values whenever suitable.*
X	☐	For Bayesian analysis, information on the choice of priors and Markov chain Monte Carlo settings
☐	☑	For hierarchical and complex designs, identification of the appropriate level for tests and full reporting of outcomes
X	☐	Estimates of effect sizes (e.g. Cohen's *d*, Pearson's *r*), indicating how they were calculated

Our web collection on statistics for biologists contains articles on many of the points above.

Software and code

Policy information about availability of computer code

Data collection	Individual files for each previously published study were collected from the contributing authors and compiled into a single dataset for each taxonomica group using publicly available packages in R version 3.6.2 and 4.0.3
Data analysis	All statistical analyses were performed in R version 4.0.3 using publicly available packages. Acompiled version of the full code used for analysis is provided in a repository at https://gitlab.com/urbionet/Trait_urban_syndromes

For manuscripts utilizing custom algorithms or software that are central to the research but not yet described in published literature, software must be made available to editors and reviewers. We strongly encourage code deposition in a community repository (e.g. GitHub). See the Nature Portfolio guidelines for submitting code & software for further information.

Data

Policy information about availability of data

All manuscripts must include a data availability statement. This statement should provide the following information, where applicable:

- Accession codes, unique identifiers, or web links for publicly available datasets
- A description of any restrictions on data availability
- For clinical datasets or third party data, please ensure that the statement adheres to our policy

All data used in this study related to taxa has been deposited in a Zenodo open data repository at https://doi.org/10.5281/zenodo.7866249. This data is publicly available. The landscape context was calculated using the publicly available Global Human Settlement (GHS) images analytics framework (http://ghsl.jrc.ec.europa.eu/ghs_bu_s1.php) and Hansen et al.'s Global Forest Change (https://glad.earthengine.app/view/global-forest-change)

Research involving human participants, their data, or biological material

Policy information about studies with human participants or human data. See also policy information about sex, gender (identity/presentation), and sexual orientation and race, ethnicity and racism.

Reporting on sex and gender	na
Reporting on race, ethnicity, or other socially relevant groupings	na
Population characteristics	na
Recruitment	na
Ethics oversight	na

Note that full information on the approval of the study protocol must also be provided in the manuscript.

Field-specific reporting

Please select the one below that is the best fit for your research. If you are not sure, read the appropriate sections before making your selection.

☐ Life sciences ☐ Behavioural & social sciences ☑ Ecological, evolutionary & environmental sciences

For a reference copy of the document with all sections, see nature.com/documents/nr-reporting-summary-flat.pdf

Life sciences study design

All studies must disclose on these points even when the disclosure is negative.

Sample size	na
Data exclusions	
Replication	
Randomization	
Blinding	

Behavioural & social sciences study design

All studies must disclose on these points even when the disclosure is negative.

Study description	na
Research sample	
Sampling strategy	
Data collection	
Timing	
Data exclusions	
Non-participation	
Randomization	

Ecological, evolutionary & environmental sciences study design

All studies must disclose on these points even when the disclosure is negative.

Study description	SEE Page 5
Research sample	
Sampling strategy	
Data collection	
Timing and spatial scale	
Data exclusions	
Reproducibility	
Randomization	
Blinding	

Did the study involve field work? ☐ Yes ☐ No

Field work, collection and transport

Field conditions	na
Location	
Access & import/export	
Disturbance	

Reporting for specific materials, systems and methods

We require information from authors about some types of materials, experimental systems and methods used in many studies. Here, indicate whether each material, system or method listed is relevant to your study. If you are not sure if a list item applies to your research, read the appropriate section before selecting a response.

Materials & experimental systems

n/a	Involved in the study	
X	☐	Antibodies
X	☐	Eukaryotic cell lines
X	☐	Palaeontology and archaeology
X	☐	Animals and other organisms
X	☐	Clinical data
X	☐	Dual use research of concern
X	☐	Plants

Methods

n/a	Involved in the study	
X	☐	ChIP-seq
X	☐	Flow cytometry
X	☐	MRI-based neuroimaging

Antibodies

Antibodies used	na
Validation	

Statistic type for inference

(See Eklund et al. 2016)

Correction

Models & analysis

n/a | Involved in the study

- [x] [ ] Functional and/or effective connectivity
- [x] [ ] Graph analysis
- [x] [ ] Multivariate modeling or predictive analysis

Functional and/or effective connectivity

Graph analysis

Multivariate modeling and predictive analysis

This checklist template is licensed under a Creative Commons Attribution 4.0 International License, which permits use, sharing, adaptation, distribution and reproduction in any medium or format, as long as you give appropriate credit to the original author(s) and the source, provide a link to the Creative Commons license, and indicate if changes were made. The images or other third party material in this article are included in the article's Creative Commons license, unless indicated otherwise in a credit line to the material. If material is not included in the article's Creative Commons license and your intended use is not permitted by statutory regulation or exceeds the permitted use, you will need to obtain permission directly from the copyright holder. To view a copy of this license, visit http://creativecommons.org/licenses/by/4.0/

Field-specific reporting

Please select the one below that is the best fit for your research. If you are not sure, read the appropriate sections before making your selection.

☐ Life sciences ☐ Behavioural & social sciences ☒ Ecological, evolutionary & environmental sciences

For a reference copy of the document with all sections, see nature.com/documents/nr-reporting-summary-flat.pdf

Ecological, evolutionary & environmental sciences study design

All studies must disclose on these points even when the disclosure is negative.

Study description	This study collated existing data from previously published studies related to community level species data from sampling sites within urban areas to investigate functional trait and diversity change with increasingly urban landscapes globally across six different taxa groups (Amphibians, Bats, Bees, Carabid beetles, Reptiles and Birds).
Research sample	The research sample consisted of 5,302 species from 72,086 plots spread across 379 cities worldwide and retained six taxonomic groups with sufficient data for a global assessment of urbanisation effects (see Fig. 1 and Supplementary Tables 1 and 2): amphibians (140 species, 1,202 plots in 191 cities), bats (84 species, 540 plots in 43 cities), bees (486 species, 471 plots in 25 cities), carabid beetles (327 species, 889 plots in 17 cities), reptiles (98 species, 321 plots in 71 cities) and birds (4,167 species, 68,558 plots in 177 cities). Data were contributed by participants, and initially cleaned and compiled by taxa coordinators before being aggregated into single data matrices (species x site; species x trait; site x environment) for each taxa group (See Supplementary Data Tables 1-6)
Sampling strategy	The sampling sizes used reflected the distribution of studies that met the inclusion criteria at the time when this research study commenced (March 2016- April 2017). Potential datasets were initially identified using a systematic literature search (See Brodsky-Rega et al. Urban Ecosystems (Accepted 24 January 2022)), personal networks of initial coauthor group and an open call via UrBioNet and presentations at INTECOL 2017 and other conferences. Authors of existing studies were contacted and invited to participate in this study under the conditions outlined in the UrBioNet Authorship Policy (https://sites.rutgers.edu/urbionet/about/authorship-guidelines/)
Data collection	Due to the large number of potential contributors globally and the complexity of working across 6 taxanomic groups we employed a "tracks and signals" system for data collection and all subsequent stages of the research process. Each taxonomic group was assigned a Taxa Coordinator group who made the initial approach to corresponding authors of the potential datasets, and managed the communication with the data contributors for that taxa group, the cleaning of the contributed data up to the point of collation into the single taxa dataset, and the population of any missing trait data. These taxa streams represent the "tracks", with the coordinators overseeing their individual line. Coordination across all lines was overseen by the project lead (AKH) who acted as the signal master and coordination communication across all taxa groups, and took ultimate responsibility for recording contributions of all participants under the Authorship Policy conditions. Taxa coordinators were: Amphibians/Reptiles (AH-M, IM-F, SK), Bats (CGT, KJ), Bees (CGT, JSM, NSW), Carabids (JK, MM), Birds (CAL, CCR-B, FAL, IMF, TS).Following initial cleaning and data compilation, the individual study files were passed on to the Data Compilation and Analysis team (FAL, AS, AKH, MM, JK, BF) who completed the data wrangling and analysis.
Timing and spatial scale	Collation of the included dataset occurred between November 2016 to December 2017. Due to the challenges of trying to standardize data across studies, populating data gaps related to functional traits for the taxa, and the complexities of trying to complete a highly complex analysis and manage a large team of collaborators who were all participating voluntarily on top of their substantive roles (including those acting in the role of project lead and taxa coordinators- many of whom were also early career researchers), there was limited scope to consider including additional studies after the full datasets had been compiled. Interruptions due to the COVID-19 pandemic further reduced any possibility of updating the datasets to include any additional studies. In terms of spatial scale, the data all relate to specific sampling locations where community level information had been collected by the authors of the original study. Therefore, they are highly resolved spatially, with sample sites ranging from point locations (e.g. bat traps and acoustic recorders) through to a maximum sample area of 500 m2 for bird data.
Data exclusions	Where data were available for a single city but as a time series we only included the data for one of the time periods sampled. Due to the species level analysis conducted, we had to aggregate subspecies to the species level for analysis. Similarly, all functional traits are represented as a single species-level value as our analysis was unable to support intraspecific trait ranges.
Reproducibility	The analyses conducted in this study required the use of existing packages within R software version 4.0.3. As part of our "tracks and signals" system we received feedback from the full list of collaborators at key stages (intial results, initial manuscript draft). In response to feedback received during these rounds we reviewed the data and made adjustments to the models multiple times. Each time, the model became more rigorous, but the overall results changed very little. Reproducibility is further supported through the detailed methodology provided with our submission
Randomization	Samples, organisms and contributors were organised into groups based on the focal taxa. This was to ensure that there was consistency within an individual taxonomic group, and allowed for more detailed discussion amongst experts who had a deep familiarity with the systems and literature associated with that specific taxa.
Blinding	Blinding was not appropriate for this study as we were performing a global analysis of previously published datsets

Did the study involve field work? ☐ Yes ☒ No
